# Supplementary material for: Single-cell analysis reveals that cryptic prophage protease LfgB protects Escherichia coli during oxidative stress by cleaving antitoxin MqsA
Source: Microbiol Spectr. 2024 Jan 11;12(2):e03471-23. doi: 10.1128/spectrum.03471-23 (PMC10846083; doi:10.1128/spectrum.03471-23)
Supplement: Supplemental material — Tables S1 to S3; Figures S1 to S7. [file spectrum.03471-23-s0001.docx]

**SUPPLEMENTARY INFORMATION**

**Single-Cell Analysis Reveals Cryptic Prophage Protease LfgB Protects *Escherichia coli* During Oxidative Stress by Cleaving Antitoxin MqsA**

**Laura Fernández-García^1,2^, Xinyu Gao^3,4^, Joy Kirigo^1^, Sooyeon Song^1,5,6^, Michael E. Battisti^1^, Rodolfo García-Contreras^7^, Maria Tomas^2^, Yunxue Guo^3,4,8^, Xiaoxue Wang^3,4,8^, and**

**Thomas K. Wood^1*^**

^1^Department of Chemical Engineering, Pennsylvania State University, University Park, Pennsylvania, 16802-4400, USA

^2^Microbiology Translational and Multidisciplinary (MicroTM)‐Research Institute Biomedical A Coruña (INIBIC) and Microbiology Department of Hospital A Coruña (CHUAC), University of A Coruña (UDC)

^3^Key Laboratory of Tropical Marine Bio-resources and Ecology, Guangdong Key Laboratory of Marine Materia Medica, Innovation Academy of South China Sea Ecology and Environmental Engineering, South China Sea Institute of Oceanology, Chinese Academy of Sciences, No.1119, Haibin Road, Nansha District, Guangzhou 511458, China

^4^University of Chinese Academy of Sciences, Beijing 100049, China

Departments of Animal Science^5^ and Agricultural Convergence Technology^6^, Jeonbuk National University, 587 Baekje-Daero, Deojin-Gu, Jeonju-Si, Jellabuk-Do, 54896, South Korea

^7^Departamento de Microbiología y Parasitología, Facultad de Medicina, Universidad Nacional Autónoma de México, Mexico City, Mexico

^8^Southern Marine Science and Engineering Guangdong Laboratory (Guangzhou)，No.1119, Haibin Road, Nansha District, Guangzhou 511458, China

*For correspondence. E-mail [twood@engr.psu.edu](mailto:twood@engr.psu.edu)

**Table S1.** (**A**) Lack of change in *lfgAB* transcription upon deletion of *mqsRA* using shake flasks rather than single cells during peroxide stress. H_2_O_2_ (20 mM) was added for 10 min for BW25113 (WT) vs. BW25113 Δ*mqsRA* Δ*kan* (Δ*mqsRA* Δ*kan*). (**B)** Repression of *lfgAB* in the BW25113 *mqsRA* deletion mutant during production of MqsA. MqsA was produced by 1 mM IPTG for 30 min. (**C**) Repression of *rpoS* in the BW25113 *lfgB* deletion mutant during production of LfgB. Δ*lfgB/*pCA24N and Δ*lfgB/*pCA24N‑*lfgB* were grown to a turbidity of 0.5, then 1 mM of IPTG was added for 1 hour to induce expression of *lfgB.*

**A**

| **Gene** | ***purM*** | | ***lfgAB*** | |
| --- | --- | --- | --- | --- |
| **Strain** | **WT** | **Δ*mqsRA* Δ*kan*** | **WT** | **Δ*mqsRA* Δ*kan*** |
| CT Mean | 17.8 ± 2.1 | 19.0 ± 0.6 | 20 ± 0.5 | 21.1 ± 0.8 |
| ΔCt |  |  | 2.2 | 2.1 |
| ΔΔCt |  |  |  | -0.04 ± 1.02 |
| Fold change |  |  |  | 1.03 |

**B**

| **Gene** | ***rrsG*** | | ***lfgA*** | | ***lfgB*** | |
| --- | --- | --- | --- | --- | --- | --- |
| **Strain** | **Δ*mqsRA* pCA24N** | **Δ*mqsRA* pCA24N-*mqsA*** | **Δ*mqsRA* pCA24N** | **Δ*mqsRA* pCA24N-*mqsA*** | **Δ*mqsRA* pCA24N** | **Δ*mqsRA* pCA24N-*mqsA*** |
| CT Mean | 13.1 ± 0.6 | 11.6 ± 0.4 | 22.7 ± 0.5 | 23.3 ± 1 | 24.7 ± 0.9 | 24.7 ± 0.4 |
| ΔCt |  |  | 9.6 | 11.6 | 11.6 | 13.1 |
| ΔΔCt |  |  |  | 2 ± 1 |  | 1.5 ± 0.6 |
| Fold change |  |  |  | -4 |  | -2.8 |

**C**

| **Gene** | ***rrsG*** | | ***rpoS*** | |
| --- | --- | --- | --- | --- |
| **Strain** | **Δ*lfgB* pCA24N** | **Δ*lfgB* pCA24N-*lfgB*** | **Δ*lfgB* pCA24N** | **Δ*lfgB* pCA24N-*lfgB*** |
| CT Mean | 23.4 ± 0.2 | 24.1 ± 0.7 | 24.1 ± 0.2 | 23.3 ± 0.1 |
| ΔCt |  |  | 0.7 | -0.8 |
| ΔΔCt |  |  |  | -1.5 ± 0.7 |
| Fold change |  |  |  | 2.8 |

**Table S2.** Single cell expression levels for *mqsRA* vs. wild-type in the presence of 20 mM H_2_O_2_ stress for the *lfg* operon (*lfgA* is also in **Table 1**).

| **Gene** | **Cluster** | **WT** | **Δ*mqsRA* Δ*kan*** |
| --- | --- | --- | --- |
| *lfgA* | 1 | 1.5 | -0.8 |
|  | 2 | -2.0 | 1.9 |
|  | 3 | 3.7 | 1.8 |
|  | 4 | 4.3 | 1.7 |
|  | 5 | 4.9 | 1.9 |
|  | 6 | 4.8 | 2.0 |
|  | 7 | 5.7 |  |
| *lfgB* | 1 | 1.1 | 0.5 |
|  | 2 | -0.5 | 0.01 |
|  | 3 | 4.1 | 2.6 |
|  | 4 | 4.8 | 2.8 |
|  | 5 | 4.9 | 2.9 |
|  | 6 | 5.3 | 3.8 |
|  | 7 | 5.4 |  |
| *lfgC* | 1 | 3.2 | -1.3 |
|  | 2 | -2.6 | 1.8 |
|  | 3 | 3.8 | 1.9 |
|  | 4 | 4.4 | 2.9 |
|  | 5 | 4.6 | 2.2 |
|  | 6 | 4.9 | 2.3 |
|  | 7 | 5.0 |  |
| *lfgD* | 1 | 1.8 | 1.3 |
|  | 2 | -1.9 | -0.2 |
|  | 3 | 3.1 | 1.2 |
|  | 4 | 4.0 | 1.6 |
|  | 5 | 4.2 | 1.7 |
|  | 6 | 4.3 | 1.6 |
|  | 7 | 4.8 |  |
| *lfgE* | 1 | 1.1 | -0.8 |
|  | 2 | -1.2 | 1.0 |
|  | 3 | 2.5 | 1.1 |
|  | 4 | 3.1 | 1.4 |
|  | 5 | 3.8 | 1.3 |
|  | 6 | 3.8 | 1.9 |
|  | 7 | 3.6 |  |

**Table S3.** Primers used in this study. F indicates forward primer and R indicates reverse primer. P indicates promoter, MP indicates mutant promoter.

| **Primers** | **Sequences** |
| --- | --- |
| **Probe amplification and *mqsA* cloning** | |
| *yfjY*-P-F | GATTGCGTAGCAGGGCCACTGGTATC |
| *yfjY*-P-R | CTGGCGTATGAGGTAATAAAGCGCG |
| *yfjY*-MP-F | TTGGCGGGCATGGAACTTTGATAATGACGCAAAAGAAGGTTTTAACCGCTATATCCA |
| *yfjY*-MP-R | TGGATATAGCGGTTAAAACCTTCTTTTGCGTCATTATCAAAGTTCCATGCCCGCCAA |
| *yfjY*-MP2-F | GATTGCGTAGCAGGGCCACTGGTATCTGCCGGTATCACCGACATTAACATTGACACAAACCAGGGCATTCACGTCCGCCTG |
| FAM-*yfjY*-P-F | GATTGCGTAGCAGGGCCACTGGTATC |
| pET28b-mqsA-F | TTAACTTTAAGAAGGAGATATACCATGGATGAAATGTCCGGTTTGC |
| pET28b-mqsA-R | CTCGAGTGCGGCCGCAAGCTTTTAGTGATGATGATGATGATGACGGATTTCATTCAATAGTTCTGGATGC |
| **qRT-PCR** | |
| *rrsG*-F | TATTGCACAATGGGCGCAAG |
| *rrsG*-R | ACTTAACAAACCGCCTGCGT |
| *lfgA*-F | GGATGGACCGCTTCTGTGAT |
| *lfgA*-R | CTCATTTCCGCACCATTGCC |
| *lfgB*-F | GCTGAAGCTGAAAATGGCGG |
| *lfgB*-R | TGAAATACAGGGCGCGTTTG |
| *rpoS*-F | GTGGTCTGGCGTTGCTGGACCTTATC |
| *rpoS*-R | TTCAATCGTCTGGCGAATCCACCAG |
| *purM-F* | CGGTGTTGATATTGACGCGGG |
| *purM-R* | CAGCACGGGTTCACGATATTTTTG |
| *Yfjxy-F* | TATCGCTGTCTGCCTGATTG |
| *Yfjxy-R* | TTGTGCGGTCAGTGGAAGTA |


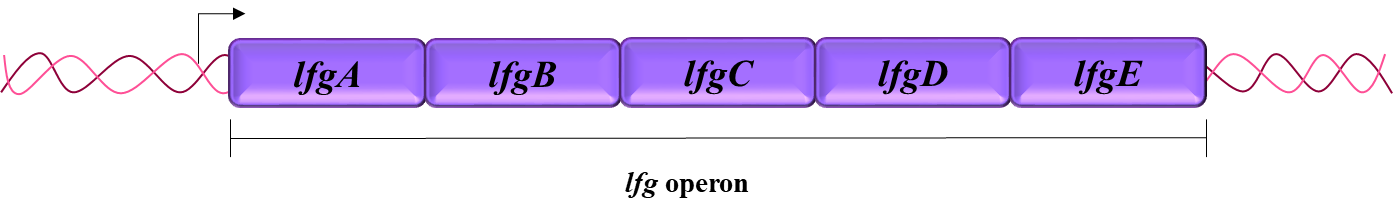


**Figure S1.** Scheme representing the organization of the *lfg* operon inside cryptic prophage CP4-57.


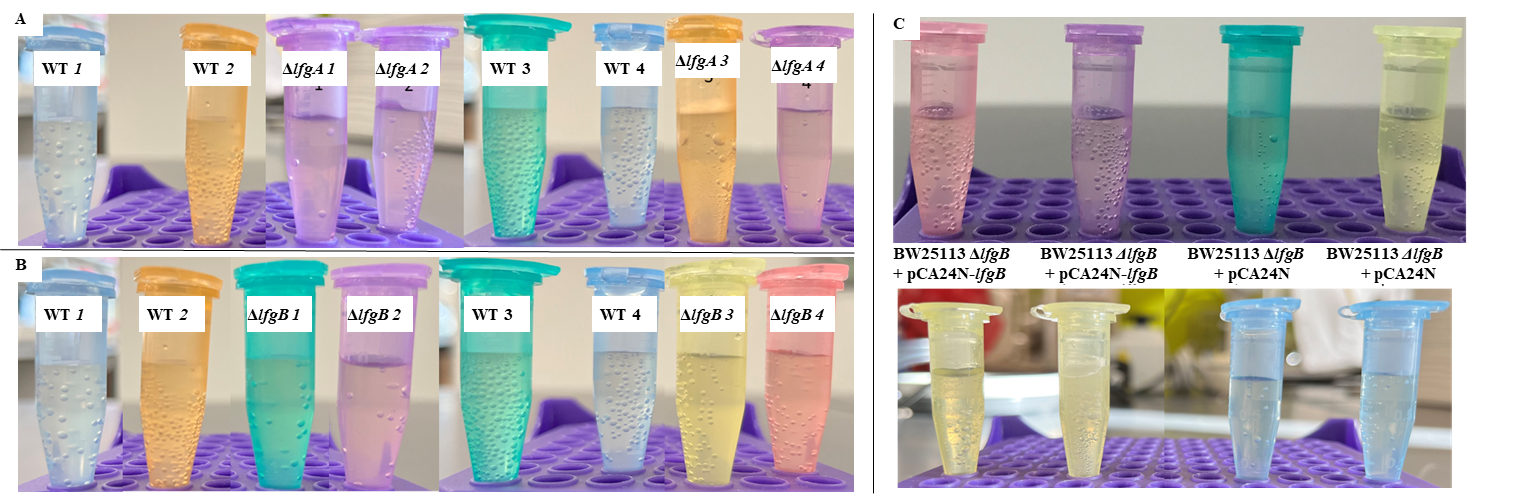


**Figure S2.** Images of oxygen bubble formation from catalase activity during the hydrogen peroxide assay for (**A**) BW25113 WT compared to BW25113 Δ*lfgA*, (**B**) BW25113 WT compared to BW25113 Δ*lfgB*, and (**C**) BW25113 pCA24N compared to BW25113 pCA24N-*lfgB*.


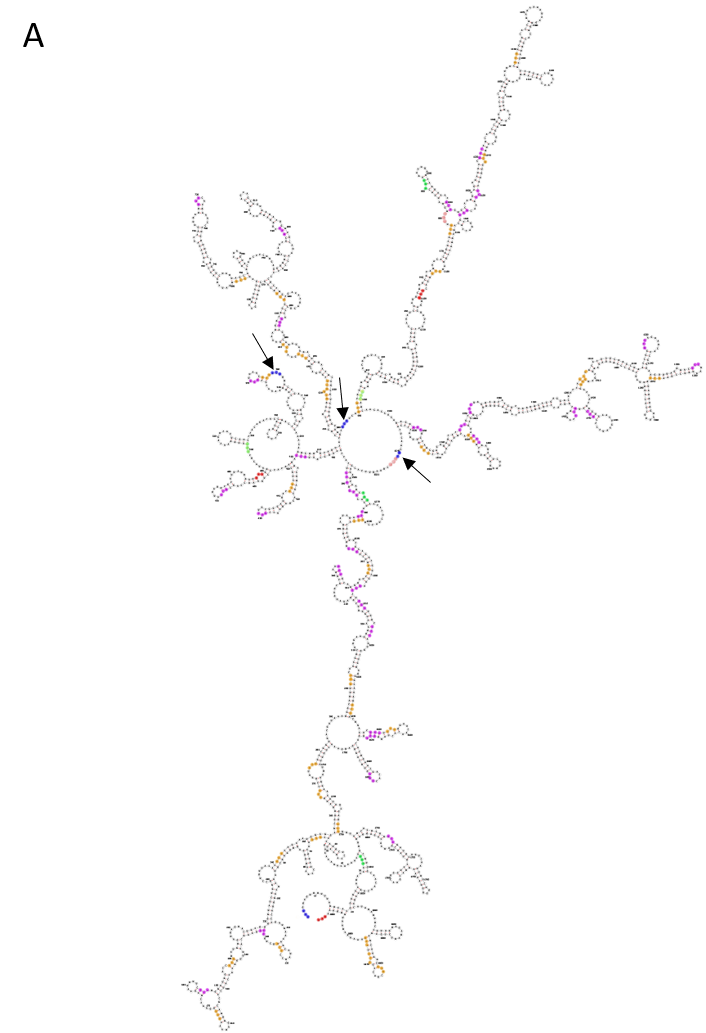


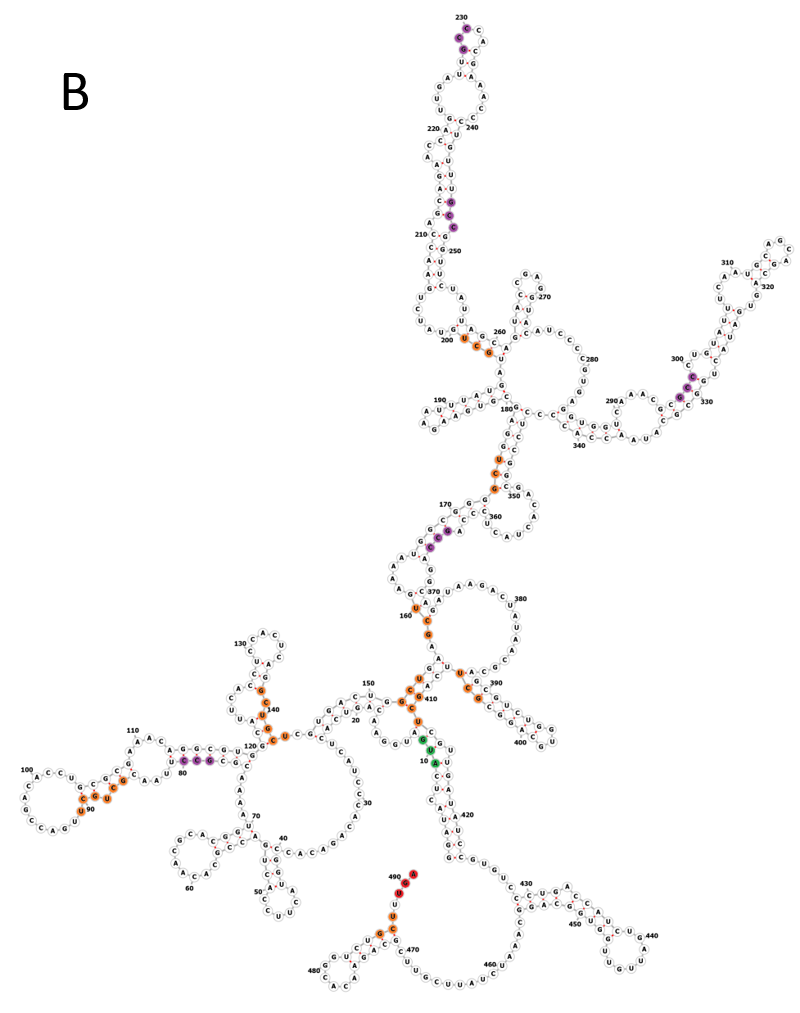


**Figure S3.** Predicted (MFE) RNA secondary structures for (**A**) the *lfg* operon and (**B**) *lfgB* indicating the presence of MqsR cleavage sites (5’-GCU). Green circles indicate start codons, red and pink circles indicate the termination codons, blue circles indicate 5’-GCU MqsR cleavage sites inside a stem-loop, purple circles indicate 5’-GCU sites outside stem-loops, and orange circles indicate 5’‑GCC sites. Arrows indicate likely MqsR single-stranded sites.

**
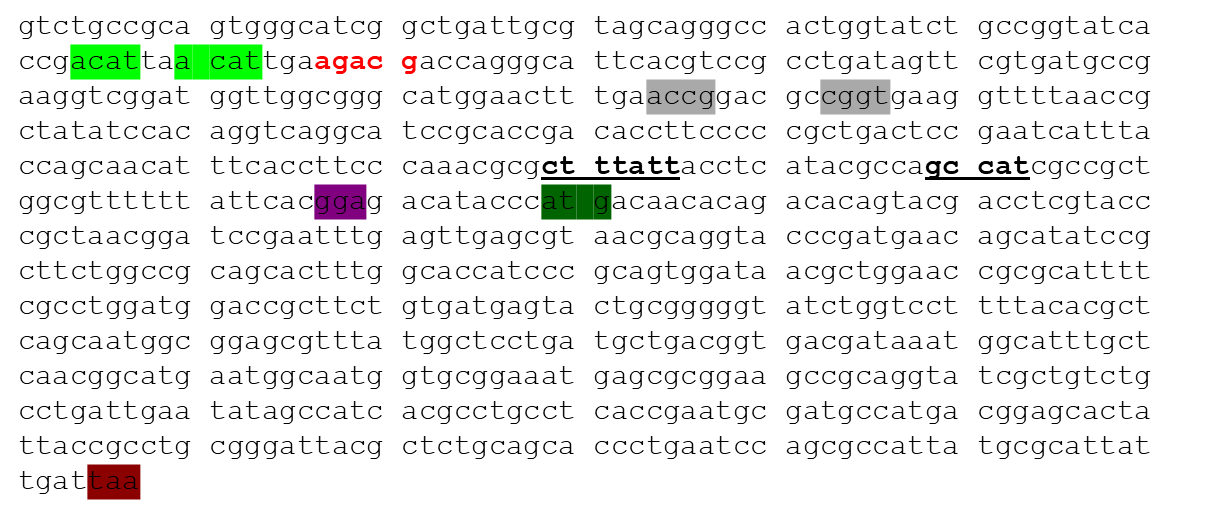
**

**Figure S4.** **Sequence of the first gene of the *lfg* operon (*lfgA*) along with the upstream region (200 bp).** MqsA-binding region identified from DNA footprinting (bold red), and previously described as 5’-ACCT N(2,6) AGGT^54^ (highlighted in gray). Bold and underlined indicates the -35 and -10 promoter regions, purple highlight indicates the ribosome binding site, and dark green highlight indicates the *lfgA* start codon. The probable binding palindrome near the DNA footprinting site is highlighted in green.

**
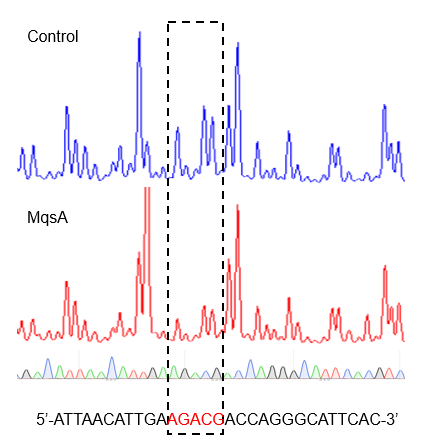
**

**Figure S5. DNA footprinting.** DNA footprinting shows the MqsA binding site is 252 bp upstream of the start codon of the *lfg* operon (bold red in **Fig. S4**), near the palindromic sequence 5’-ACAT (N2) ACAT (green highlight in **Fig. S4**).


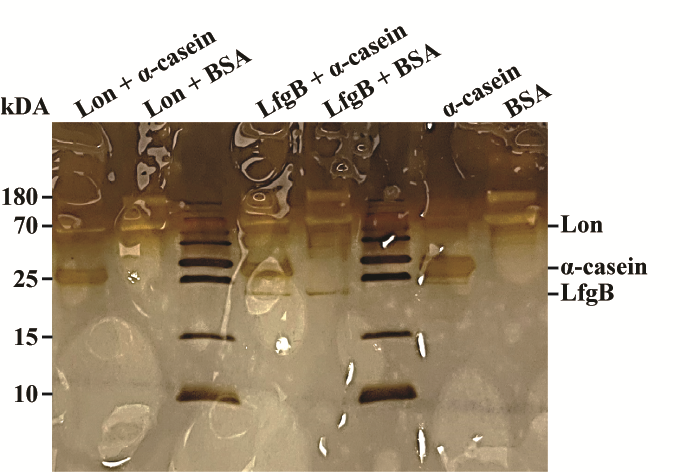


**Figure S6.**  Protease controls showing the degradation of α-casein by LfgB, LfgB alone in BSA, the degradation of α-casein by LfgB, and Lon in BSA. Purified proteins were mixed in enzyme reaction buffer and incubated for 3 hours at 37ºC.

**
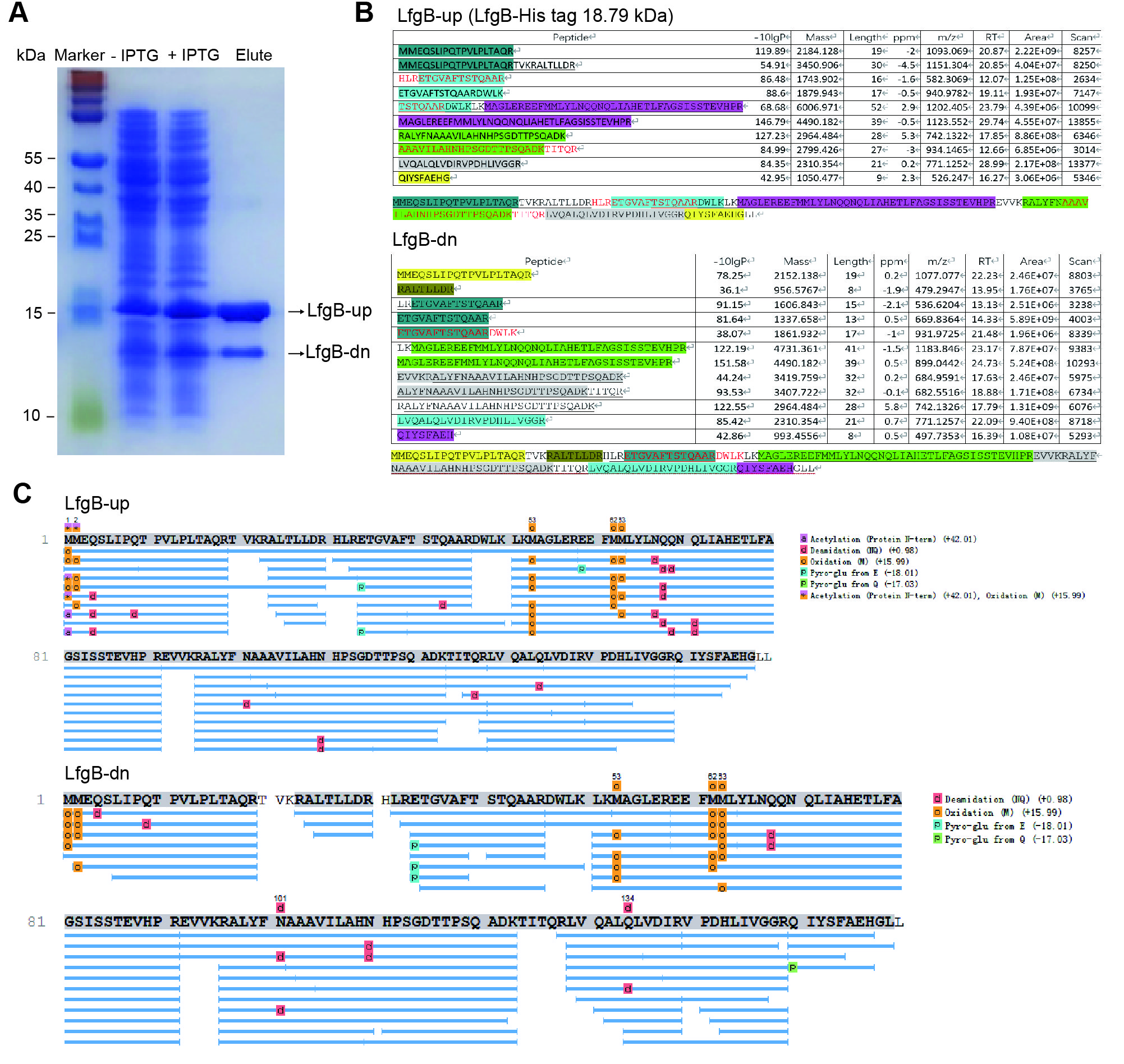
**

**Figure S7. Purification of LfgB.** (**A**) SDS-PAGE gel with the elution fraction for the purification of LfgB. (**B**) Mass-spectrometry fragments identified for the purified LfgB protein bands shown in panel **A**. (**C**) Mass spectroscopy sequence fragments for LfgB-up and LfgB-dn. LfgB-up is the full protein shown in panel **A**, and LfgB-dn is the degradation product likely due to self-digestion.
